# Twenty years of the *MEROPS* database of proteolytic enzymes, their substrates and inhibitors

**DOI:** 10.1093/nar/gkv1118

**Published:** 2015-11-02

**Authors:** Neil D. Rawlings, Alan J. Barrett, Robert Finn

**Affiliations:** 1 The Wellcome Trust Sanger Institute, Wellcome Trust Genome Campus, Hinxton, Cambridgeshire, CB10 1SA, UK; 2 EMBO European Bioinformatics Institute, Wellcome Trust Genome Campus, Hinxton, Cambridgeshire, CB10 1SD, UK

## Abstract

The *MEROPS* database (http://merops.sanger.ac.uk) is an integrated source of information about peptidases, their substrates and inhibitors, which are of great relevance to biology, medicine and biotechnology. The hierarchical classification of the database is as follows: homologous sets of sequences are grouped into a protein species; protein species are grouped into a family; families are grouped into clans. There is a type example for each protein species (known as a ‘holotype’), family and clan, and each protein species, family and clan has its own unique identifier. Pages to show the involvement of peptidases and peptidase inhibitors in biological pathways have been created. Each page shows the peptidases and peptidase inhibitors involved in the pathway, along with the known substrate cleavages and peptidase-inhibitor interactions, and a link to the KEGG database of biological pathways. Links have also been established with the IUPHAR Guide to Pharmacology. A new service has been set up to allow the submission of identified substrate cleavages so that conservation of the cleavage site can be assessed. This should help establish whether or not a cleavage site is physiologically relevant on the basis that such a cleavage site is likely to be conserved.

## INTRODUCTION

The *MEROPS* database, which is a manually curated information resource for proteolytic enzymes, their inhibitors and substrates, will be 20 years old this year. The database, which can be found at http://merops.sanger.ac.uk, was founded in 1996. There are usually three releases of the *MEROPS* database per year.

The hierarchical classification, which was established for peptidases in 1993 (1) and for peptidase inhibitors in 2004 (2), involves the clustering of homologous sets of peptidase and protein inhibitor sequences into peptidase and inhibitor species, which are in turn clustered into families, which are clustered into clans. A family contains related sequences, and a clan contains related structures. Sequence analysis is restricted to that portion of the protein directly responsible for peptidase or inhibitor activity which is termed the ‘peptidase unit’ or the ‘inhibitor unit’. The peptidase unit includes primary substrate binding sites (though not necessarily secondary binding sites, known also as ‘exosites’) and the catalytic residues. The inhibitor unit is a domain that interacts with a peptidase domain and, if one exists, will include the reactive bond that occupies the active site. A peptidase or inhibitor unit normally corresponds to a structural domain, and some proteins contain more than one peptidase or inhibitor domain. Examples are potato virus Y polyprotein which contains three peptidase units, each in a different family, and turkey ovomucoid, which contains three inhibitor units all in the same family. At every level in the database a well-characterized type example is nominated, to which all other members of the family or clan must be shown to be related in a statistically significant manner. The type example at the peptidase or inhibitor level is termed the ‘holotype’ (1,2). Criteria for distinguishing one peptidase species from another were established in 2007 (3).

Each clan, family, holotype peptidase and holotype inhibitor is assigned to an identifier. For a clan, the identifier consists of two letters, the first of which indicates the catalytic type (‘A’ for aspartic peptidase, ‘C’ for cysteine peptidase, ‘G’ for glutamic peptidase, ‘M’ for metallopeptidase, ‘S’ for serine peptidase, ‘T’ for threonine peptidase and ‘N’ for asparagine lyase). For simplicity, we here use the term ‘peptidase’ for any proteolytic enzyme, although a few of them are not peptidases in the strictest sense because they are lyases and not hydrolases (4). There are three additional letters: ‘P’ for peptidases of mixed catalytic type, ‘U’ for peptidases of unknown catalytic type and ‘I’ for inhibitors that are proteins. An example of a clan identifier is AA which includes aspartic peptidases with a pepsin-like fold. For a family, the identifier consists of an initial letter, again corresponding to catalytic type, and a number. An example is A1, the family of pepsin-like aspartic peptidases. For a holotype, the identifier consists of the family name (padded with a zero when necessary to make it three characters long), a dot and a number. An example is cathepsin D: A01.009. An identifier where ‘9’ follows the dot is a non-peptidase homologue (e.g. pregnancy-associated glycoprotein 1, A01.971). An identifier where ‘P’ follows the dot is a pseudogene (e.g. the napsin B pseudogene, A01.P01).

Among the criteria for distinguishing one peptidase from another is the action on substrates. A collection of known cleavage sites in substrates, including proteins, peptides and synthetic substrates, has been established (5). Similarly, a collection of peptidase-inhibitor interactions has also been established, which provides evidence for distinguishing peptidases and inhibitors (6). Because the MEROPS classification of inhibitors can only be applied to inhibitors that are proteins, a second, unclassified, collection of small molecule inhibitors was established (6).

In addition, the MEROPS database and website includes an extensive, manually-selected bibliography. References are assigned to the relevant MEROPS identifiers so that there are references for each clan, family, peptidase, inhibitor, substrate cleavage and peptidase-inhibitor interaction.

Statistics from release 9.13 (July 2015) of *MEROPS* are shown in Table 1 and compared with release 9.9 from August 2013. Counts of substrate cleavages, peptidase-inhibitor interactions and references are shown in Table 2.

**Table 1.** Counts of protein species, families and clans for proteolytic enzymes and protein inhibitors in the *MEROPS* database

	MEROPS 9.9		MEROPS 9.13	
	Peptidases	Inhibitors	Peptidases	Inhibitors
Sequences	413 834	28 502	523 871	74 658
Identifiers (Total)	4384	712	4622	712
experimentally characterized and sequenced	2638	593	2794	597
hypothetical from model organisms	1352	0	1628	0
not active as peptidase or inhibitor	324	115	333	115
experimentally characterized but unsequenced	203	0	203	0
pseudogenes	70	0	70	0
Compound and complex proteins	16	49	16	56
Families	244	76	253	79
Clans	55	39	61	39

The numbers in Release 9.13 of *MEROPS* (July 2015) are compared to those in Release 9.9 of *MEROPS* (August 2013). A peptidase is referred to as ‘unsequenced’ when no sequence is known, or the known sequence fragments are insufficient to be able to assign the peptidase to a family.

**Table 2.** Information in the MEROPS database

	MEROPS 9.9	MEROPS 9.13
Substrate cleavages: total	61 357	64 470
Substrate cleavages: physiological	16 580	17 131
Substrate cleavages: non-physiological	35 364	36 981
Substrate cleavages: pathological	1235	1426
Substrate cleavages: synthetic substrates	5235	5965
Peptidase-inhibitor interactions: total	4475	6202
Peptidase-inhibitor interactions: proteins	1373	1428
Peptidase-inhibitor interactions: SMI	2790	4419
References	52 600	59 155

Substrate cleavage totals do not include cleavages derived only from the SwissProt database (mainly removal of initiating methionines and signal peptides). A naturally occurring cleavage is described as ‘physiological’ when the peptidase and substrate are from the same organism, and ‘pathological’ if the organisms differ and are pathogen and host.

There are other Internet resources available for peptidases. The following databases and/or websites provide information about post-translational processing events: the CutDB database and its associated PMAP platform provide physiological substrate cleavage data and pathways and networks for proteolytic systems (7,8); TOPPR (The Online Protein Processing Resource) is a database for cleavages identified by the COFRADIC proteomics method (9); TopFind (and its associated tools TopFinder and PathFinder) identifies mature N- and C-termini in proteins from six model organisms (10); DegraBase provides information on N-termini of mature human proteins identified by proteomics (11); and Proteasix is a tool to help predict the human peptidase responsible for peptide production, based on peptides identified from human urine (12). Specialist resources for particular peptidase families include CaspDB, a collection of predicted caspase cleavage sites in the human proteome (13); MerCASBA, a collection of known caspase substrates (14); and CleavPredict, a collection of predicted matrix metallopeptidase cleavage sites in the human proteome (15). The services POPS and PROSPER provide insights into peptidase specificity and predictions of cleavages in substrates, respectively (16,17).

### Changes to existing features and methodologies

#### Model organisms

Every peptidase in the proteome of a model organism is assigned to a MEROPS identifier. If the homologue is apparently not the orthologue of a known, characterized peptidase, then a special identifier is established in which the character after the dot is a letter ‘A’, ‘B’ or ‘C’. The zebrafish (*Danio rerio*) has been added to the list of model organisms in *MEROPS* and a new *MEROPS* identifier has been created for each peptidase that could not be assigned to an existing identifier. The full list of model organisms in *MEROPS* is: human, mouse, zebrafish, the fruit fly *Drosophila melanogaster*, the nematode *Caenorhabditis elegans*, mouse-ear cress (*Arabidopsis thaliana*), baker's yeast (*Saccharomyces cerevisiae*), the fission yeast *Schizosaccharomyces pombe*, the malaria parasite *Plasmodium falciparum*, the slime mould *Dictyostelium discoideum*, the Gram-negative bacterium *Escherichia coli*, the Gram-positive bacterium *Bacillus subtilis* and the archaean *Pyrococcus furiosus*.

#### Corrections to sequences, peptidase units and active site residues

All the sequences of peptidase homologues have been subjected to an automated comparison with the domains as defined in the Pfam database (18; http://pfam.xfam.org). The consequences of this comparison have been: (i) to identify and remove any false positives that were filed because they matched domains other than the peptidase domain; (ii) to further refine the extent of peptidase units so that sequence that is part of a domain other than the peptidase domain is excluded; (iii) to update any sequence with one that has been modified since it was filed in *MEROPS*; and (iv) to recalculate active site residues and metal ligands with improved algorithms. In total 16 319 sequence records in *MEROPS* have changed, and a considerable number of former non-peptidase homologues have been reclassified as peptidases.

#### *MEROPS* identifier assignment

The method for assigning a *MEROPS* identifier is derived from the phylogenetic tree: an identifier is assigned to all sequences derived from the same node as a holotype. There are, however, problems with this approach, including the time taken to generate all the alignments and trees, and the method was not applied to non-peptidase homologues and fragments because these were excluded from the alignments and thus also from the trees. We have adopted an alternative methodology to help with the assignment of *MEROPS* identifiers. A sequence library is generated for each family and FastA (19) is used to search this library with each holotype sequence from the same family. Each hit is sorted to the holotype with the lowest E value and given the same *MEROPS* identifier, provided the sequence identity is 40% or more. To remove fragments and sequences that are near duplicates a program has been written to perform pairwise comparisons with FastA and merge the sequences if they are greater than 94% identity, from the same species, not known to be products of different genes and not tandem duplicates in the same genome.

#### Non-peptidase homologues

A new routine to calculate active site residues in genome-derived sequences has been introduced. This has enabled many sequences formerly considered to be fragments or non-peptidase homologues to be reclassified as peptidases. A library of non-peptidase homologues in FastA format is generated and then subject to search with each of the holotypes. From the pairwise match the active site residues and/or metal ligands are recalculated, and if a match is found in which all essential residues are conserved, then the annotation in the database is updated with the new predictions and the homologue reclassified as a peptidase.

#### Small molecule inhibitors

The number of small molecule inhibitors included in the database has been increased (see Table 2). Previously, only those with a full summary were included. The small molecule inhibitor pages have been modified so that they conform more to the style used in other pages. There are buttons across the top of each page for some or all of the following: Summary, Structure, References and Inhibits. The Structure page lists all the Protein Data Bank (20) entries that include complexes with a peptidase for the inhibitor in question. Rows in the table are ordered by the peptidase inhibited. The columns in the table are identical to the structure pages for peptidases and protein inhibitors. The table in the Inhibits page lists the *MEROPS* identifier for the peptidase and its recommended name, the name of the inhibitor, the inhibition constant (*K*_i_) for complex formation (if known), conditions under which inhibition occurs and a reference. The table can be re-ordered by clicking the column heading.

The corresponding Inhibitors page for a peptidase now includes all the relevant small molecule inhibitors. Where possible, the small molecule inhibitor name includes a link to the relevant small molecule inhibitor summary.

#### Change to MEROPS identifiers index

We have decided that the expressed sequence tag (EST) analyses have become less useful with the developments in genome sequencing and visualization and localization techniques. The counts of EST sequences have been removed from the MEROPS identifiers index page for peptidases and replaced by counts of substrate cleavages.

#### Alignments and trees

It has been apparent for some time that the full family and subfamily alignments and trees are in many cases unusable because there are so many sequences and the aligned sequences are too long. We have decided that for any family or subfamily with more than 200 sequences then only a representative set will be shown. The representative set includes all family or subfamily holotypes as well as an example from every organism phylum where a member species has a homologue that is predicted to be an active peptidase or peptidase inhibitor. The format of the alignments and trees remains the same. Because we have to generate full alignments and trees to assign *MEROPS* identifier correctly, the full alignments and trees which are not being displayed on the website are made available to download from our FTP site. We have added options on each family summary page to download the library of peptidase unit sequences (in FastA format), the aligned sequences (also in FastA format) and the tree (in Newick format, http://evolution.genetics.washington.edu/phylip/newicktree.html).

#### References

A new reference topic for localization (or visualization) has been introduced to bibliographies. The full list of categories is as follows:


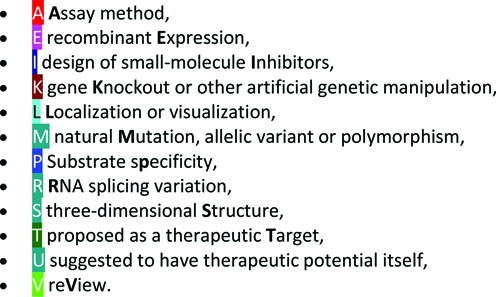


On any reference list for clan, family, peptidase or inhibitor, the list of topics is shown and by clicking on a topic the list is filtered for references pertinent to that topic.

### Recent developments

#### Pathways

There are many physiological pathways that involve peptidases and their inhibitors, such as the blood coagulation cascade, assembly of complement, control of blood pressure and apoptosis. There are many high quality web services that display biological pathways, and rather than attempt to generate our own displays, we have made cross-links to the KEGG database for biological pathways (21). The cross-links can be seen on any peptidase or inhibitor summary page. The section headed ‘Pathway’ lists all the KEGG pathways in which the peptidase or inhibitor is involved, and on clicking the name of the pathway the user will be sent to the relevant pathway map in the KEGG database.

**Figure 1.**
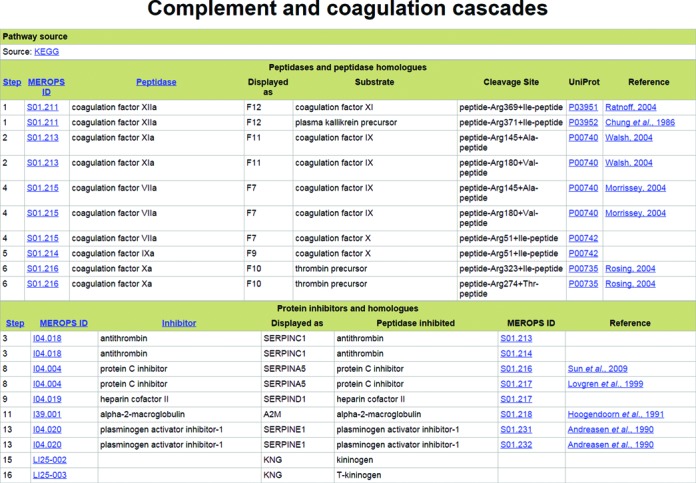
An example of a biological pathway page. The page displaying peptidases and peptidase inhibitors involved in the blood coagulation and complement activation pathway is shown. Only part of each table is shown.

We are in a unique position, thanks to our collections of cleavages in physiological substrates and peptidase-inhibitor interactions, to add extra information for each biological pathway involving peptidases. We have created a page for each pathway, and an example pathway page is shown in Figure 1. The cross-link to the KEGG database is displayed, along with two tables.

The first table shows the peptidases involved in the pathway and the columns are: a pathway step number; the MEROPS identifier for the peptidase, which is a link to the peptidase summary page; the recommended name for the peptidase; how the peptidase is displayed on the KEGG pathway scheme; the name of a substrate cleaved by the peptidase and relevant to the pathway; a description of the cleavage site in the substrate with ‘+’ indicating the cleavage site; the UniProt accession for the substrate (which is a cross-link to the substrate page in *MEROPS*); and a reference for the cleavage. What is shown as a single event on a KEGG pathway schema may represent one or more cleavage events. There are some pathways in which peptidases are involved but their action on a substrate is either not known or the cleavage position is not known, or the peptidase is acting in a process that does not require its proteolytic activity. In these cases, a cleavage cannot be shown.

The second table shows the peptidase inhibitors involved in the pathway and the columns are: the pathway step; the MEROPS identifier for the inhibitor, which is a link to the inhibitor summary page; the recommended name for the inhibitor; how the inhibitor is displayed on the KEGG pathway scheme; the name of a peptidase inhibited by the inhibitor and relevant to the pathway; the MEROPS identifier for the peptidase, which is a link to the peptidase summary page; and a reference describing the peptidase-inhibitor interaction. As described above for a peptidase, there may be some process involving a peptidase inhibitor which does not require its inhibitory activity, and under such circumstances a peptidase-inhibitor interaction cannot be shown.

We should like to point out that many cleavage events essential to a pathway are generally not shown on pathways schemas. These include intracellular processing events such as removal of a signal peptide, an initiating methionine or some other transit peptide.

The pathway step is an arbitrary number created by us, generally numbering each occurrence of a peptidase or peptidase inhibitor from the top left to the bottom right of the KEGG pathway scheme. The pathway step is used internally to link the pathway to a cleavage and a peptidase-inhibitor interaction.

An index to all KEGG pathways in which peptidases or peptidase inhibitors are known to be involved has been provided as an item on the opening, left hand green menu on the *MEROPS* website. On clicking a pathway name, a pathway page is displayed.

Peptidases and peptidase inhibitors are involved in 205 of the pathways displayed in KEGG. These pathways involve 325 peptidases, 41 peptidase inhibitors and 309 different cleavages in substrates. Comparing these numbers with Tables 1 and 2, it is clear that most peptidases and most physiological peptidase cleavages have not yet been included in pathways.

#### Links to the IUPHAR guide to pharmacology

Cross-links have been made between peptidases and protein inhibitors in *MEROPS* to the IUPHAR Guide to Pharmacology (22). These can be found on the relevant Pharma pages. There are 55 peptidases and seven peptidase inhibitors linked to 65 IUPHAR identifiers.

#### Analysis of substrates

**Figure 2.**
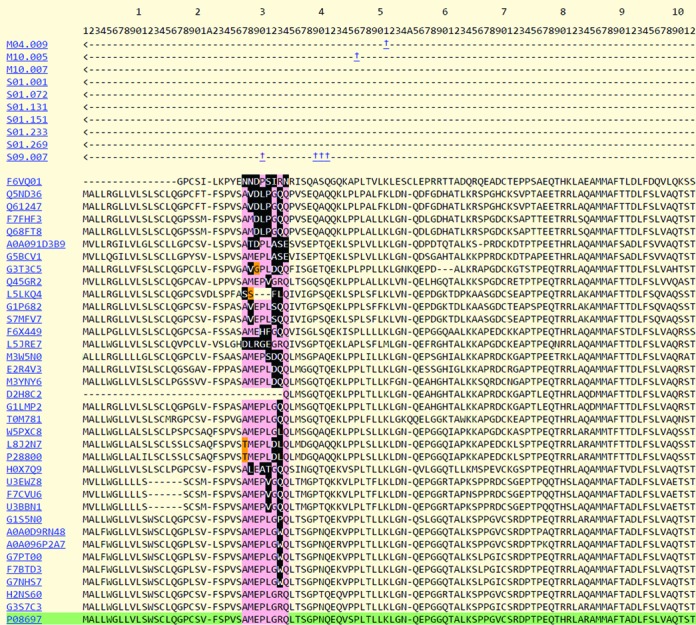
An example of results returned by the substrate analysis service. Alignment of homologues of alpha-2-antiplasmin showing cleavage at residue 30 by fibroblast activation protein alpha subunit (S09.007). This is the fifth result from the list shown above. The known cleavage occurs in the human protein (UniProt: P08697; 27) and is highlighted in green. Four residues either side of the cleavage site are highlighted. A conserved residue is highlighted in pink. A residue that differs from that in the human sequence is highlighted in orange if the same amino acid is known to occupy the same binding site from another substrate of this peptidase; and in black if the amino acid is not known to occur in the same binding site from any substrate for this peptidase. The high number of residues highlighted in black indicates that this cleavage is unlikely to be physiologically relevant, but note that the number of cleavages known for this peptidase is low.

A new service to analyse protein substrate cleavages has been introduced. This service gives an indication of whether the cleavage site is conserved amongst orthologous sequences. This service is primarily intended for bulk upload of substrate cleavages detected by high-throughput methods where it is difficult to distinguish cleavages that are physiologically relevant from those that occur but have no physiological significance (‘bystander’ substrates) and anomalous cleavages that occur because the normal barriers that prevent cleavage have been removed, for example in a cell lysate. The user can upload a tab-delimited file containing on each line the MEROPS identifier of the peptidase responsible for the cleavage, the Uniprot accession of the substrate, and the residue number (from the Uniprot entry) where cleavage occurs (for an example, see Table 3a). Homologues of this substrate are downloaded from the UniRef50 database (23) and aligned using MUSCLE (24). The residues P4-P4’ around the cleavage site (in the nomenclature of Schechter & Berger (25)) are scored according to whether they are conserved with the known substrate, and whether a replacement amino acid is known in that particular binding pocket from all known substrates for the peptidase in question. An amino acid that it not known to occupy that particular binding pocket in any of the substrates of the peptidase is termed an ‘unacceptable replacement’. A cleavage site containing many unacceptable replacements is unlikely to be derived from a physiological substrate of the peptidase, assuming that for a physiologically relevant substrate the cleavage site would be conserved. A poorly conserved cleavage site is either not physiologically relevant, or represents a pathological cleavage restricted to one or a few organisms. Results are returned by E-mail. An example of the results returned is shown in Table 3b. By clicking on the URL, the user is able to view the alignment in question. In Table 3b, it is immediately apparent that the first two cleavage sites are well conserved and that there are no unacceptable replacements. These are likely to be physiological cleavages. There are two unacceptable replacements in P4 for the cleavage of mouse ADAMTS-1 (UniProt: P97857) by furin (S08.071) at residue 251 where in both cases serine is replaced by asparagine in the orthologues from sheep and the Chines tree shrew (*Tupaia chinensis*). These replacements are probably insignificant, and the furin cleavage is most likely also physiologically relevant. Cleavage of alpha-2-antiplasmin (UniProt: P08697) at residue 128 by aureolysin (M04.009), a secreted metalloendopeptidase from *Staphylococcus aureus*, is unlikely to be physiological because of the larger number of unacceptable replacements in several substrate binding pockets. The last cleavage, of alpha-2-antiplasmin at residue 30 by fibroblast activation protein alpha subunit (S09.007), also has large numbers of unacceptable replacements in all eight positions P4-P4’ and might also be assumed to be an unlikely physiological event. The alignment in Figure 2 shows orthologues of alpha-2-antiplasmin from the wide variety of species contained in the relevant UnIRef50 entry: and the large number of residues highlighted in black around the cleavage site is immediately obvious. These are the unacceptable replacements. However, it should be noted that the number of substrates known for fibroblast activation protein alpha subunit is small (6, see column headed ‘total cleavages’ in Table 3b, compared with aureolysin for which there are 24 known substrate cleavages). This probably indicates that too little is known about substrate preferences for the peptidase to be able to draw any firm conclusions. The Analyse Substrates service is described in more detail in another paper (26).

**Table 3a.** Examples of input and output from the Analyse Substrates service. a) A table showing data for submission to the Analyse Substrates service. The user is asked to supply a plain text file with elements separated by tabs. There should be one cleavage per line, and each cleavage should consist of the MEROPS identifier of the peptidase performing the cleavage, the UniProt accession of the protein substrate, and the residue number after which cleavage occurs (the P1 residue). Residue numbering should be taken from the relevant UniProt entry. Column headings (shown here for guidance only) are not required

*MEROPS* identifier	UniProt accession	cleaved at
C01.034	O15144	68
M10.003	O15144	208
S08.071	P97857	251
M04.009	P08697	398
S09.007	P08697	30

**Table 3b.** A table of results from a submission to the substrate analysis service. The table shows the MEROPS identifier for the peptidase (submitted by the user); the total number of cleavages in all substrates for this peptidase; the UniProt accession of the protein substrate (submitted by the user); the P1 position in the cleavage (submitted by the user); the number of mismatches from an alignment of homologues from the same UniRef50 entry as the submitted substrate for positions P4 to P4’; and a URL linking to the same alignment on the *MEROPS* website.

MEROPS ID	total cleavages	UniProt Acc	homologues in UniRef50	cleavage	P4	P3	P2	P1	P1’	P2’	P3’	P4’	URL
C01.034	756	O15144	32	68	0	0	0	0	0	0	0	0	URL
M10.003	3417	O15144	32	208	0	0	0	0	0	0	0	0	URL
S08.071	208	P97857	83	251	2	0	0	0	0	0	0	0	URL
M04.009	24	P08697	128	398	50	1	31	31	49	0	31	2	URL
S09.007	6	P08697	128	30	46	57	53	53	60	29	64	13	URL

The Analyse Substrates service is designed for submission of many cleavage sites in substrates and the results returned are designed to be easily parsed. To view the conservation of a single cleavage site, or even to view a site that is entirely theoretical, the reader is reminded that this is possible by adjusting the parameters associated with the URL. In a URL for a substrate alignment, for example http://merops.sanger.ac.uk/cgi-bin/align_substrate?acc=P01966;mid=A01.009;residue=25 (cleavage of cattle haemoglobin alpha subunit by cathepsin D at residue 25), the parameters are UniProt accession (acc), *MEROPS* identifier (mid) and residue number at which cleavage occurs (residue). By changing these parameters a user can view any cleavage in any alignment by any peptidase.
